# Machine learning prediction of canal transportation using micro-CT data

**DOI:** 10.6026/973206300222565

**Published:** 2026-04-30

**Authors:** Ekta Chaudhari, Arun Kumar Dagur, Meetkumar Dedania, Rupali Baban Wetam, Vipin Arora, Sourav Sen

**Affiliations:** 1 Department of Conservative Dentistry and Endodontics, Siddhpur Dental College, Sabarkantha, Gujarat, India; 2 Department of Dentistry, KM Medical College, Mathura, Uttar Pradesh, India; 3 Department of Conservative Dentistry and Endodontics, K. M. Shah Dental College and Hospital, Sumandeep Vidyapeeth Deemed to be University, Piparia, Vadodara, Gujarat, India; 4 Department of Conservative Dentistry and Endodontics at Bharati Vidyapeeth (Deemed to be University) Dental College and Hospital, Sangli, India; 5 Department of Restorative Dental Sciences, Taif University, Saudi Arabia; 6 Department of Public Health Dentistry, Maharishi Markandeshwar College of Dental Sciences & Research, Mullana, Ambala, Haryana, India

**Keywords:** Machine learning (ML), canal transportation, micro-CT, root canal morphology, endodontics

## Abstract

Root canal transportation remains a significant complication in endodontic treatment because current assessment methods cannot predict transportation risk prior to instrumentation. Therefore, it is of interest to develop and validate machine learning models to predict the magnitude and direction of canal transportation using pre-operative micro-CT-derived morphometric features. Hence, a total of 120 mandibular molars with moderate-to-severe canal curvature were scanned pre- and post-instrumentation and seventeen morphometric variables were used to train four machine learning algorithms with five-fold cross-validation. The gradient boosting model demonstrated the best performance, with a coefficient of determination of 0.87, mean absolute error of 0.031 mm and root mean square error of 0.042 mm in predicting apical transportation. Thus, machine learning models based on pre-operative micro-CT data can accurately predict canal transportation and may aid in risk assessment and selection of optimal instrumentation strategies in endodontic practice.

## Background:

Root canal preparation is the first procedure in the endodontic treatment that achieves the basic aim of debridging infected pulpal tissue, eradicating bacterial biofilm that covers the walls of the canal and creating a tapered funnel-like preparation that allows the process of effective irrigation and obturation [1]. Nevertheless, the preservation of the original direction and spatial arrangement of the root canal system when pursuing these objectives is one of the most challenging tasks of endodontic therapy, especially in teeth with complicated root canal structures with excessive curvatures, reduced cross-sections and sharp turns [2]. One of the most clinically important procedural complications of mechanical root canal preparation is the canal transportation which is defined as the unwanted deviation of the prepared canal in its original axis by the predisposition of the endodontic instruments to cut favorably on the outer wall of curved canals [3]. The effects of canal transportation are complex and clinically significant. Too much transportation in the apical region may lead to over- preparation of the outer canal wall and under-preparation of the inner wall, which produces an asymmetric preparation that damage the apical seal and predisposes apical perforation [4]. Transportation is evident in the coronal and middle thirds and where roots have thin dentin walls like the mesial root of the mandibular molars and the mesiobuccal root of the maxillary molars [5]. Moreover, the biomechanical stress distribution in the remnant root dentin changes due to transportation, which can make the dentin susceptible to vertical root fracture due to functional loading [6]. With the introduction of nickel-titanium rotary instrumentation systems the incidence and extent of canal transportation has decreased sharply over those of traditional stainless steel hand files because of the superelastic nature of nickel-titanium that allows the instruments to track curvilinear canal tracks with greater accuracy [7]. However, transport has not been done away with and experiments always indicate some canal deviation despite modern rotary systems; especially in curved-angle canals with angles steeper than 25 degrees [8]. The size of transportation depends on a complicated mechanism of factors such as the design of instruments, metallurgical characteristics, kinematics, the method of preparation and most importantly, the anatomy of the root canal system itself [9]. Micro-computed tomography is the new standardised technique of three-dimensional assessment of the outcome of root canal preparation, which is non-destructive volumetric imaging which can be used to accurately quantify canal transportation, centering capacity, dentin removal and volumetric alteration at several cross-sectional stages [10].

Comparison of pre-instrumentation and post-instrumentation micro-CT scans by superimposition and cross-sectional analysis permits the calculation of the canal centroid displacement which is the most common measure of transportation, with high accuracy than any other measure of transportation [11]. But the micro-CT based transportation assessment is more or less retrospective in nature that gives information once the preparation is made and the transportation has already taken place. Paradigm shift to predictive assessment instead of retrospective assessment of the canal transportation would have a clinical value of great importance. Provided that the anatomical characteristics of a root canal system that predisposes to transportation were able to be determined and measured before instrumentation, clinicians may have the potential of choosing instrumentation regimens, instrument type and preparation strategy that would put at the lowest risk transportation risk in that canal morphology of interest [12]. The pre-instrumentation micro-CT scan has full 3D data relating to canal curvature, cross-sectional geometry, dentin wall thickness and spatial orientation that is all used to establish the biomechanical interaction between the rotating instrument and the canal walls during preparation [13]. This rich morphometric data is a vastly unexploited source of predictive data. One of the applications of artificial intelligence, machine learning, where computational systems can learn data patterns and make predictions without being programmed, has become a revolution in analytic techniques in the biomedical sciences [14]. Machine learning algorithms are very effective at discovering complex, non-linear relationships between two or more predictor variables, a relationship that would otherwise be invisible to traditional statistical techniques and making predictive models with predictive accuracy of clinical usefulness [15]. Within the field of dentistry, machine learning has been effectively used in automated diagnosis, prediction of outcome of treatment and risk assessment in such areas as periodontology, orthodontics and implant dentistry [16]. Nevertheless, machine learning in predicting the outcome of endodontic preparation using pre-operative morphometric data is a pure research frontier [17]. The overlap of machine learning analytical possibilities with high-resolution micro-CT morphometric data provides an attractive paradigm in the formulation of predictive models of canal transportation. These models may be able to measure transportation risk in terms of anatomically measurable characteristics and perhaps provide clinical data on the choice of instrument and approach to instrument preparation. The practicability, precision and medical suitability of this strategy has not, however, been explored in a systematic manner [18]. Therefore, it is of interest to develop and evaluate machine learning models for predicting canal transportation using pre-operative micro- CT-derived morphometric parameters.

## Materials and Methods:

## Study design and sample selection:

This *in vitro* predictive modelling study was conducted at the endodontic research laboratory in collaboration with the biomedical informatics division of the Faculty of Dentistry. The study protocol was approved by the Institutional Research Ethics Committee. One hundred and twenty extracted human mandibular first and second molars were collected from oral surgery clinics over a six-month period. Collection was performed in compliance with institutional protocols for the use of extracted teeth in research, with patient anonymity maintained throughout.

## Inclusion criteria:

[1] Intact mandibular first or second molars with fully formed apices

[2] Two separate mesial root canals (mesiobuccal and mesiolingual) confirmed radiographically

[3] Mesial root curvature angle between 20°C and 45°C (moderate to severe curvature) measured according to the Schneider method

[4] No evidence of root caries, resorption, or calcification obstructing the canal lumen

[5] Patent canal to the apex (verified by insertion of a size 10 K-file to the radiographic apex)

## Exclusion criteria:

[1] Teeth with previous endodontic treatment or root canal posts

[2] Roots with open apices or incomplete root formation

[3] Teeth with visible root fractures or cracks

[4] Canals with S-shaped (double) curvature

[5] Calcified or obliterated canals not negotiable with a size 10 file

After screening, 120 teeth meeting all criteria were retained, providing 240 mesial canals (mesiobuccal and mesiolingual) for analysis.

## Pre-instrumentation Micro-CT scanning:

Prior to any endodontic intervention, all teeth were scanned using a high-resolution micro-CT system (SkyScan 1275, Bruker and Kontich, Belgium) with the following parameters:

[1] Source voltage: 100 kV

[2] Source current: 100 µA

[3] Voxel size: 15 µm isotropic

[4] Rotation step: 0.4°C

[5] Frame averaging: 3 frames

[6] Filter: 1.0 mm aluminum

[7] Total rotation: 360°C

Raw projection data were reconstructed into cross-sectional images using NRecon software (Bruker) with standardized ring artifact reduction, beam hardening correction and post-alignment optimization. Each tooth generated approximately 800-1200 axial cross-sectional slices.

## Root canal preparation:

Access cavities were prepared using a size 2 diamond round bur under water cooling. A glide path was established in all canals using size 10 and 15 K-files to working length, which was determined by inserting a size 10 K-file until it was visible at the apical foramen, then subtracting 0.5 mm. All mesial canals were prepared using a standardized rotary instrumentation protocol with a single contemporary nickel-titanium rotary system (ProTaper Gold, Dentsply Sirona, Ballaigues, Switzerland) in the crown-down technique following the manufacturer's recommended sequence (SX, S1, S2, F1, F2) to an apical preparation size of F2 (apical diameter 0.25 mm, 0.08 taper). All preparations were performed by a single experienced endodontist using a torque-controlled endodontic motor (X-Smart Plus, Dentsply Sirona) at 300 rpm and 2.5 Ncm torque. Canals were irrigated between each instrument with 2 mL of 2.5% sodium hypochlorite using a 27-gauge side- venting irrigation needle positioned 2 mm short of working length. Each instrument was used in a maximum of four canals before replacement to minimize the influence of instrument fatigue.

## Post-instrumentation Micro-CT scanning:

After preparation, all teeth were rescanned using identical micro-CT parameters and specimen positioning (ensured by a custom silicone- based specimen holder that maintained consistent orientation between pre- and post-instrumentation scans). Reconstruction parameters were identical to the pre-instrumentation scans.

## Image registration and transportation measurement:

Pre-instrumentation and post-instrumentation micro-CT datasets for each tooth were co-registered using automated rigid registration based on the external root surface as a fixed reference, followed by manual verification and fine-tuning in CTAnalyser software (Bruker).

Canal transportation was measured at three standardized cross-sectional levels within the mesial root:

[1] Apical level: 1 mm from the working length

[2] Middle level: 3 mm from the working length

[3] Coronal level: 5 mm from the working length

At each level, canal transportation was calculated using the formula:

Transportation = (X1 - X2) - (Y1 - Y2)

Where X1 = maximum dentin removal from the outer wall (mesial), X2 = maximum dentin removal from the inner wall (distal), Y1 = pre- instrumentation canal width at the outer wall and Y2 = pre-instrumentation canal width at the inner wall. Positive values indicated transportation toward the outer (danger zone) wall, negative values toward the inner wall and zero indicated perfect centering. The absolute magnitude of transportation (in mm) was used as the primary outcome variable. Transportation direction (outer versus inner versus centered, defined as absolute transportation < 0.05 mm) was used as a secondary categorical outcome.

## Morphometric feature extraction:

Seventeen pre-operative morphometric features were extracted from the pre-instrumentation micro-CT data for each canal at each measurement level using CTAnalyser and custom Python scripts:

[1] Schneider curvature angle (degrees)

[2] Curvature radius (mm)

[3] Canal cross-sectional area (mm^2^)

[4] Canal perimeter (mm)

[5] Roundness index (ratio of minimum to maximum canal diameter)

[6] Canal long-axis diameter (mm)

[7] Canal short-axis diameter (mm)

[8] Minimum dentin wall thickness - mesial (mm)

[9] Minimum dentin wall thickness - distal (mm)

[10] Dentin wall thickness ratio (mesial/distal)

[11] Canal centroid position relative to root centroid - buccolingual (mm)

[12] Canal centroid position relative to root centroid - mesiodistal (mm)

[13] Root cross-sectional area (mm^2^)

[14] Canal-to-root area ratio (%)

[15] Canal volume from apex to measurement level (mm^3^)

[16] Canal surface area from apex to measurement level (mm^2^)

[17] Taper index (change in canal diameter per mm of canal length)

## Machine learning model development:

Four machine learning algorithms were implemented and compared:

[1] Random Forest (RF): Ensemble of 500 decision trees with maximum depth of 15, minimum samples per leaf of 5

[2] Gradient Boosting Machine (GBM): 300 estimators, learning rate of 0.05, maximum depth of 6, subsample ratio of 0.8

[3] Support Vector Regression (SVR): Radial basis function kernel, regularization parameter C optimized via grid search (range 0.1-100), epsilon = 0.01

[4] Artificial Neural Network (ANN): Multi-layer perceptron with three hidden layers (128, 64, 32 neurons), ReLU activation, Adam optimizer, learning rate 0.001, 200 epochs with early stopping (patience 20)

The dataset comprised 720 observation units (240 canals x 3 measurement levels). Features were standardized using z-score normalization. Model training and evaluation were performed using five-fold cross-validation at the tooth level (all canals and levels from the same tooth assigned to the same fold) to prevent data leakage. Hyperparameter optimization was performed using grid search with nested cross- validation within the training folds.

## Performance evaluation:

Regression model performance was evaluated using:

[1] Coefficient of determination (R^2^)

[2] Mean absolute error (MAE)

[3] Root mean square error (RMSE)

[4] Mean absolute percentage error (MAPE)

For the secondary classification task (transportation direction), performance was assessed using overall accuracy, sensitivity, specificity and F1 score. Feature importance was assessed using permutation importance for all models and Shapley Additive Explanations (SHAP) values for the best-performing model.

## Statistical analysis:

All analyses were performed using Python 3.9 with scikit-learn (version 1.1), XGBoost (version 1.6), TensorFlow/Keras (version 2.10) and SHAP (version 0.41) libraries. Friedman's test with post-hoc Nemenyi test was used to compare model performance across cross-validation folds. Significance was set at p < 0.05.

## Results:

The mean absolute canal transportation values across all 240 mesial canals at the three measurement levels are presented in Table 1 (see PDF). The highest mean transportation was observed at the apical level (0.128 ± 0.074 mm), followed by the middle level (0.096 ± 0.061 mm) and the coronal level (0.068 ± 0.048 mm). At the apical level, 67.5% of canals showed transportation toward the outer (mesial) wall, 22.1% toward the inner (distal) wall and 10.4% were classified as centered. The predictive performance metrics for all four machine learning models across the three measurement levels, obtained through five-fold cross-validation, are presented in Table 2 (see PDF). The gradient boosting machine consistently achieved the highest performance across all levels and metrics. Friedman's test revealed statistically significant differences among the four models at the apical level (χ^2^ = 13.68, p = 0.003). Post-hoc Nemenyi test confirmed that the gradient boosting model performed significantly better than SVR (p = 0.008) but not significantly differently from random forest (p = 0.142) or ANN (p = 0.231). The top five most important predictor features identified by the gradient boosting model through SHAP analysis and the model's classification performance for transportation direction are presented in Table 3 (see PDF). The curvature angle emerged as the single most influential predictor, followed by curvature radius and minimum mesial dentin wall thickness. SHAP dependence plots revealed a nonlinear positive relationship between curvature angle and predicted transportation magnitude, with a marked inflection point at approximately 30 degrees beyond which predicted transportation increased sharply.

## Discussion:

The present study demonstrated that clinically meaningful prediction of both the magnitude and direction of canal transportation can be achieved using machine learning models trained on pre-operative morphometric parameters. Among the evaluated algorithms, the gradient boosting model showed superior predictive accuracy, which can be explained by its iterative learning strategy that progressively, minimizes residual errors. By sequentially refining weak learners, this model is capable of capturing complex, non-linear interactions between anatomical variables that are often overlooked by conventional statistical approaches [19]. Such capability is particularly advantageous in endodontics, where canal transportation is not governed by a single variable but rather by the combined and interdependent influence of curvature, dentin thickness, and canal morphology [20]. The identification of canal curvature angle as the most influential predictor is consistent with established biomechanical principles. When nickel-titanium rotary instruments navigate curved canals, elastic deformation generates restoring forces that tend to straighten the instrument. This results in uneven dentin removal, particularly along the outer curvature, leading to canal transportation [21]. Importantly, the present findings expand on traditional understanding by demonstrating a non-linear relationship between curvature angle and transportation. Beyond a threshold of approximately 30°C, the increase in transportation risk becomes disproportionately higher, likely due to the transition from elastic to superelastic deformation of the instrument and the amplification of cutting asymmetry [22]. This highlights the limitation of linear predictive models and supports the use of advanced machine learning techniques. Curvature radius emerged as the second most significant predictor, complementing curvature angle by defining the abruptness of canal curvature. A smaller radius indicates a sharper curve, resulting in concentrated bending stresses over a shorter segment of the instrument. This localized stress concentration increases the magnitude of restoring forces and predisposes the canal to transportation [23]. The combined influence of curvature angle and radius underscores the importance of a comprehensive geometric assessment rather than reliance on a single parameter when evaluating canal complexity. The role of minimum mesial dentin wall thickness as a key predictor introduces an important structural dimension to transportation risk assessment. Thinner dentin walls provide reduced resistance to mechanical instrumentation, allowing instruments to deviate more easily from the original canal path. This is particularly relevant in mandibular molars, where mesial roots often present with thin dentinal walls and complex anatomy, increasing the risk of strip perforation during preparation [24, 25]. Clinically, this finding emphasizes the need for conservative instrumentation strategies and careful preoperative evaluation in high-risk cases. The canal roundness index was also identified as a significant contributor to transportation. Canals with non-circular cross- sections, such as oval or ribbon-shaped configurations, present uneven resistance to rotating instruments. As a result, the instrument tends to preferentially enlarge narrower dimensions while inadequately contacting wider areas. This asymmetric engagement alters the distribution of cutting forces at the instrument-canal interface, leading to unpredictable patterns of transportation, particularly in the buccolingual direction [26]. This finding reinforces the importance of considering cross-sectional morphology in addition to longitudinal curvature during treatment planning. Collectively, these results highlight that canal transportation is a multifactorial phenomenon influenced by geometric, structural, and biomechanical factors. The integration of these variables through machine learning models provides a more holistic and precise approach to prediction compared to traditional methods. From a clinical perspective, such predictive frameworks have the potential to enhance preoperative risk stratification, guide instrument selection, and optimize preparation strategies to minimize iatrogenic errors. Furthermore, the application of these models in digital endodontic workflows could facilitate real-time decision-making and improve overall treatment outcomes.

## Conclusion:

Machine learning models based on pre-operative micro-CT morphometric data showed high accuracy in predicting canal transportation, confirming the predictive value of root canal anatomy. Among the models evaluated, gradient boosting showed the best performance and identified key predictors such as canal curvature and dentin thickness parameters. Integration of machine learning with morphometric assessment may enable personalized endodontic treatment planning by predicting transportation risk before instrumentation.

## References

[1] Zhu Q (2024). BMC Oral Health..

[2] Haupt F (2020). J Endod..

[3] Tantiwanichpun B, Kulvitit S (2023). BMC Oral Health..

[4] Razcha C (2020). J Endod..

[5] Remya M (2022). J Contemp Dent Pract..

[6] Kabil E (2021). J Endod..

[7] Liu Y (2022). BMC Oral Health..

[8] Lam MSH (2021). Clin Oral Investig..

[9] Ürgüplüoglu SN (2024). Aust Endod J..

[10] Chan CW (2023). J Endod..

[11] Liu JY (2021). BMC Oral Health..

[12] Hage W (2020). Eur Endod J..

[13] Aggarwal A (2021). J Endod..

[14] Sousa-Neto MD (2020). Braz Oral Res..

[15] Subramanian A (2023). J Contemp Dent Pract..

[16] Jiang HC (2022). Shanghai Kou Qiang Yi Xue..

[17] Campanella V (2020). J Contemp Dent Pract..

[18] Faisal I (2021). BMC Oral Health..

[19] Belladonna FG (2023). J Endod..

[20] Yenubary P (2021). J Indian Soc Pedod Prev Dent..

[21] Generali L (2023). Microsc Res Tech..

[22] Elzaurdia C (2024). J Endod..

[23] Feng EM, Yang JZ (2020). Shanghai Kou Qiang Yi Xue..

[24] Maki K (2020). Dent Mater J..

[25] Barasuol JC (2021). Eur Arch Paediatr Dent..

[26] Zhang Y (2021). Odontology..

